# Targeting macrophage-myofibroblast transition with *Caulis spatholobi* to attenuate renal interstitial fibrosis: integrated UHPLC-Q-Exactive Orbitrap-MS, network pharmacology, and experimental validation

**DOI:** 10.3389/fphar.2025.1649902

**Published:** 2025-10-09

**Authors:** Ziyi Song, Yunlong Zhang, Chao Yang, Kexin Ren, Yijing Cheng, Zhujiang Zhang, Tianjiao Ren, Yixuan Chen, Xue Li, Yan Lin

**Affiliations:** ^1^ School of Stomatology, Qiqihar Medical University, Qiqihar, China; ^2^ School of Public Health, Qiqihar Medical University, Qiqihar, China; ^3^ School of Medical Technology, Qiqihar Medical University, Qiqihar, China; ^4^ School of Basic Medicine, Qiqihar Medical University, Qiqihar, China; ^5^ Heilongjiang Provincial Key Laboratory of Medicine-Food Homologous Resources and Metabolic Disease Prevention and Control, Qiqihar Medical University, Qiqihar, China; ^6^ Postdoctoral Research Station of Qiqihar Pharmaceutical Sciences Research Institute, Qiqihar, China

**Keywords:** renal interstitial fibrosis, *Caulis spatholobi*, network pharmacology, molecular docking, macrophage-to-myofibroblast transition

## Abstract

**Introduction:**

*Caulis Spatholobi* (CS), a traditional Chinese medicine, is recognized for its abilities to reduce fibrinogen levels, promote proteolysis, and improve conditions such as diabetic nephropathy. However, the potential of aqueous extract of CS (AECS) as an effective treatment for renal interstitial fibrosis (RIF) is yet to be established.

**Methods:**

The AECS was qualitative analyzed by UHPLC-Q-Exactive Orbitrap-MS. Potential targets of AECS were predicted, and RIF disease targets were collated from databases. A Venn diagram was generated using the EVenn platform, and drug-active ingredient-target network diagrams were constructed with Cytoscape 3.10.1 software. The PPI network was generated through the STRING database, and GO and KEGG enrichment analyses were executed via the DAVID platform. Molecular docking predictions of active ingredients binding with core targets were conducted using the CB-Dock2 platform. Finally, the anti-RIF effect of AECS was evaluated in an adenine-induced rat model.

**Results:**

A total of 64 chemical constituents were identified in the AECS. 97 common targets for treating RIF were identified through mining multiple databases. These key targets, particularly AKT1, EGFR and IL6, mediated biological functions such as protein phosphorylation and regulated several signaling pathways, including PI3K/Akt. Molecular docking studies demonstrated that ingredients like licochalcone A exhibited strong binding affinity with hub genes such as AKT1, EGFR and IL6. In an RIF rat model, treatment groups showed reduced renal tissue damage. Furthermore, treatment with AECS significantly ameliorated renal dysfunction in RIF rats, along with a downregulation of RIF markers α-SMA and fibronectin. Compared to the AECSL group, the LST and AECSH groups (300mg/kg/d) exhibited more significant therapeutic effects. Ultimately, RIF model rats showed increased expression of pan-macrophage marker CD68 and M2-specific marker CD206, along with α-SMA co-expression, indicating differentiation into MMT cells displaying CD68^+^α-SMA^+^ or CD206^+^α-SMA^+^ immunophenotypes. LST and AECS treatments significantly reduced MMT cell populations, with CD206^+^α-SMA^+^ cells being more abundant than CD68^+^α-SMA^+^ cells, emphasizing the key role of M2 macrophages in MMT-driven RIF. MMT-derived M1-like cells secreted IL-6 while M2-like cells produced IL-10, AECS downregulated both cytokines.

**Conclusion:**

Our study is expected to provide the pharmacological mechanisms by which CS may be a promising anti-RIF drug for future clinical trials.

## Graphical Abstract

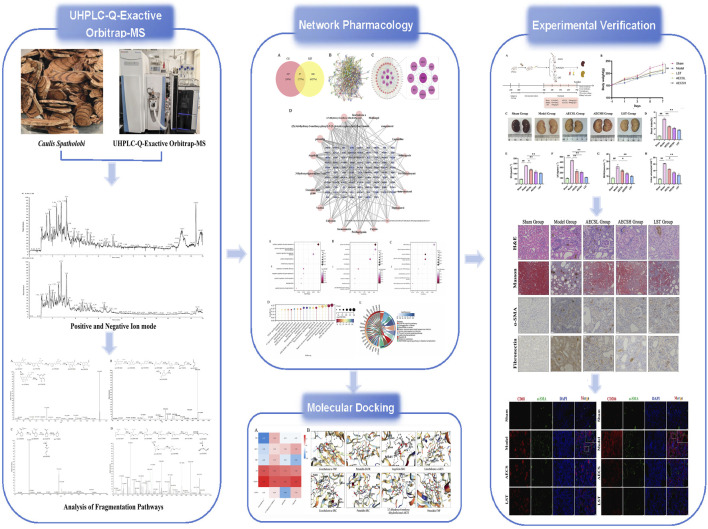

## 1 Introduction

The global incidence of chronic kidney disease (CKD) is increasing and presents an urgent public health concern (Jie et al., 2023). It affects 8%–16% of the global population (Kovesdy, 2022). Since 1990, both the prevalence and mortality rates of CKD across all age groups have risen by 29.3% and 41.5% (Li J. C. et al., 2024), respectively, with expectations of continued growth, significantly endangering human health. The pathogenesis of CKD is intricate, with renal interstitial fibrosis (RIF) identified as a primary pathological feature. RIF is characterized by glomerulosclerosis, renal tubular atrophy, and interstitial fibrosis, and is recognized as the final pathway leading to end-stage renal disease, or renal failure (Ren et al., 2024). The high cost of treatment, coupled with limited options such as dialysis or kidney transplantation, and numerous complications, significantly impairs patients’quality of life (Thurlow et al., 2021). Recent studies have indicated that the abnormal expression of various pro-inflammatory factors, cytokines, growth factors, chemokines, and acute-phase proteins results in the destruction of the renal tubular basement membrane, atrophy and collapse of tubular epithelial cells, widening of the interstitial space, extensive deposition of extracellular matrix (ECM), and increased infiltration of inflammatory cells, along with the transformation of resident cells (Isaka, 2018)^.^ Additionally, the regulation of the degradation enzyme system is weakened (Kessenbrock et al., 2010), leading to increased expression of collagen and fibronectin (FN), structural remodeling of renal tissue, and the progression of RIF. Recent studies demonstrated that macrophages played a pivotal role in chronic kidney disease (CKD) progression (Xia et al., 2025). These cells were differentiated into pro-inflammatory M1 macrophages and anti-inflammatory M2 macrophages, with the latter primarily contributing to macrophage-to-myofibroblast transition (MMT). Interleukin-6 (IL-6) was identified as a classical pro-inflammatory cytokine, whereas interleukin-10 (IL-10) functioned as a typical anti-inflammatory factor. MMT cells were characterized by coexpression of the macrophage markers CD68 (cluster of differentiation 68) or CD206 (mannose receptor C-type 1), along with the myofibroblast marker α-smooth muscle actin (α-SMA). Both *in vitro* and *in vivo* experiments (Sinha et al., 2025; Zhao et al., 2025) revealed that MMT inhibition effectively attenuated collagen deposition and RIF progression, likely through the indirect downregulation of IL-6 and IL-10.

Currently, the primary clinical treatments for RIF predominantly involve the use of immunosuppressive agents, glucocorticoids, and diuretics, yet there remains a notable absence of specific therapeutic interventions targeted specifically at RIF. *Caulis spatholobi* (CS), the dried vine stem of the legume plant *Spatholobus suberectus* Dunn, is documented in the ancient text *Bencao Beiyao* as possessing properties that “promote blood circulation and relieve tendon stiffness, treat both male and female dry blood fatigue, and serve as a vital medicinal remedy for various deficiencies and ailments.” Modern pharmacological research has revealed that CS exhibits a broad spectrum of biological activities, including anti-tumor, immunomodulatory, antibacterial, antioxidant, fibrinogen-lowering, and pro-proteolytic effects (Ban et al., 2023; Wu et al., 2024). It has been discovered that the ethanol extract of CS demonstrates antiplatelet activity by inhibiting the binding of fibrinogen to GPⅡb/Ⅲa receptors, resulting in reduced mortality in thromboembolic model mice (Lee et al., 2011). Furthermore, Liu W. et al. (2024), have identified that the aqueous extract of CS (AECS) significantly lowers serum creatinine and blood urea nitrogen levels, restores organ indices, and improves renal pathological conditions in hyperuricemic mice. Nevertheless, whether CS modulated MMT to affect RIF progression, and its potential as a therapeutic target for RIF, had not been investigated, and the underlying mechanisms remained to be elucidated.

Network pharmacology is a research area that analyzes the active ingredients in prescriptions from a systemic perspective, elucidating their potential mechanisms. This approach closely aligns with the holistic view of traditional Chinese medicine (TCM) and the core principle of syndrome differentiation and treatment, and it has been validated as a more appropriate method for analyzing the multiple ingredients, targets, and pathways of TCM (Wang et al., 2021). Molecular docking is a technique utilized to predict the binding affinity of ligand compounds to proteins with known three-dimensional structures (Wang and Li, 2023). This study investigates the potential active ingredients, core action targets, and possible mechanisms of CS in treating RIF using UHPLC-Q-Exactive Orbitrap-MS and network pharmacology. In addition, molecular docking techniques are employed, and a rat model of RIF is established to further validate the core targets and the efficacy of the AECS in treating RIF, thereby providing new insights and directions for drug development and clinical applications.

## 2 Materials and methods

### 2.1 CS action target collection

Using “*Caulis Spatholob*” as a keyword, effective ingredients were screened from the Traditional Chinese Medicine Systems Pharmacology Database and Analysis Platform (TCMSP, http://tcmspw.com/tcmsp.php) based on criteria of oral bioavailability (OB) ≥ 30% and drug-likeness (DL) ≥ 0.18. The SMILES numbers of the effective ingredients were acquired from the PubChem database (https://pubchem.ncbi.nlm.nih.gov/). In the Swiss Target Prediction database (http://swisstargetprediction.ch/), with “*Homo sapiens*” specified as the species, the corresponding targets and gene names of the effective ingredients were documented.

### 2.2 RIF disease target collection

Using OMIM (http://www.omim.org/), STRING database (http://string-db.org/), GENE) CARDS (https://www.genecards.org/), DisGeNET (http://www.disgenet.org/), and DrugBank (https://www.drugbank.ca/) databases, the keyword “Renal interstitial fibrosis” was entered to retrieve targets associated with RIF. These targets were confirmed and converted using the UniProt database (https://www.uniprot.org/), and duplicates were removed from the integrated list. By employing the EVenn platform (http://www.ehbio.com/test/venn), drug-related targets were mapped with disease-related targets to identify potential key targets for CS in the treatment of RIF. A Venn diagram was then drawn to visualize these overlaps using the EVenn platform (http://www.ehbio.com/test/venn).

### 2.3 “Drugs-active ingredient-target” visualization network construction

The active pharmaceutical ingredients and key targets obtained from the screening were matched. Utilizing Cytoscape 3.10.1 software (https://cytoscape.org/), the relationships were visualized, leading to the construction of a“drugs-active ingredient-target”network pharmacology diagram.

### 2.4 Construction of protein-protein interaction (PPI) network

The key targets were imported into the STRING database v11.0 (https://string-db.org/), and a PPI network was constructed with species limited to “*Homo Sapiens*,” yielding the protein interaction relationships. The resultant data were then imported into Cytoscape v3.10.1 for visual analysis, resulting in the construction of a PPI network diagram. In this network, node size and color intensity indicated the size of degree, while edge thickness and color intensity indicated the size of combined score.

### 2.5 GO and KEGG pathway enrichment analysis

The key intersection targets were uploaded to the DAVID platform (https://david.ncifcrf.gov/) for Kyoto Encyclopedia of Genes and Genomes (KEGG) enrichment analysis and Gene Ontology (GO) functional enrichment analysis, with species limited to “*H. sapiens*.” GO encompassed three domains: Biological Process (BP), Cellular Component (CC), and Molecular Function (MF). Pathway analysis focused on KEGG, with results filtered for FDR <0.1 and *P* < 0.05.

### 2.6 Molecular docking analysis and visualization

The top 4 targets with the highest Degree in the PPI network were selected for molecular docking analysis. The corresponding target protein 3D structures were downloaded from the PDB database (https://www.rcsb.org/) and uploaded to the CB-Dock2 platform (https://cadd.labshare.cn/cb-dock2/php/index.php) for further computational and analytical simulation docking processing, yielding docking data. Binding activity was evaluated based on the binding energy: a binding energy below −5.0 kcal/mol typically indicated good binding activity between the donor and receptor, while a binding energy below −7.0 kcal/mol indicated stronger binding activity (Dong et al., 2021). The results were then subjected to visual analysis.

### 2.7 UHPLC-Q-Exactive Orbitrap-MS analysis of AECS chemical constituents

#### 2.7.1 Preparation of aqueous extract of CS

*Caulis spatholobi* was procured from Beijing Tongrentang Herbal Pieces Co.,Ltd. (20160274) and authenticated by professor Tingting Liu of the Qiqihar Medical University. Approximately 1000 g of raw *Caulis spatholobi* herb was weighed and ground into powder. This powder was soaked in 1,000 mL of distilled water for 30 min, then heated to maintain a gentle boil for 45 min. The mixture was filtered, and the residue was extracted with additional distilled water for another 30 min. The two filtrates were combined. The combined filtrates were concentrated to 1 g/mL using a 100 °C water bath, and the resultant solution was stored at 0–5 °C prior to subsequent use.

The AECS was precisely measured to 1 mL using an electronic analytical balance (Shanghai, China). The extract was mixed with 10 mL of chromatographic-grade methanol via vortexing, followed by ultrasonic-assisted extraction for 15 min in an ultrasonic cleaning machine (Yunnan, China; 400 W, 40 kHz). After cooling to room temperature, the mixture was centrifuged at 8,000 rpm for 10 min using a 1–14 high-speed microcentrifuge (Sigma, Germany). The resulting supernatant was collected, filtered through a 0.22 μm microporous membrane, and transferred to an autosampler vial for subsequent analysis using a Thermo UHPLC-Q-Exactive Orbitrap-MS spectrometer (UltiMate 3,000, MA, United States).

#### 2.7.2 UHPLC-Q-Exactive Orbitrap-MS qualitative analysis

Chromatographic separation was performed on a Waters ACQUITY UPLC BEH C18 Column (100 mm × 2.1 mm, 1.7 μm). The mobile phase was composed of phase A (0.1% formic acid aqueous solution) and phase B (0.1% formic acid–acetonitrile solution). The gradient elution protocol used for the analysis was as follows: 0–12.0 min, 5% B→95% B; 12.0–12.9 min, 95% B; 12.9–13.0 min, 95% B→5% B; 13.0–15.0 min, 5% B.

The electrospray ionization source (ESI source) was operated in both positive and negative ion monitoring modes (ESI^+^, ESI^−^) using full MS/ddMS2 scanning. Key parameters were set as follows: sheath gas flow rate at 9 arb, spray voltage at 3 kV, capillary temperature at 320 °C, S-lens voltage at 55 kV, and auxiliary gas heating temperature at 30 °C. Full MS scanning was performed over a range of m/z 100-1,200 with a resolution of 70,000, while maintaining 1e6 ions in the C-Trap and a maximum injection time of 100 ms. For ddMS2 mode, the resolution was set to 35,000 with an apex trigger time of 3–9 s, collision energies of 20, 40, and 60 kV, and a dynamic exclusion time of 8 s.

Based on the precise molecular weight information obtained from mass spectrometry, molecules were selected when the deviation between the measured and theoretical mass-to-charge ratios of their primary quasi-molecular ion peaks was less than 3 ppm. These molecules were then matched against a self-constructed database of drug chemical components. By integrating the characteristic fragmentation patterns of secondary mass spectrometry and neutral loss rules, the chemical identities were inferred.

### 2.8 *In vivo* validation experiment

#### 2.8.1 Experimental grouping and dosing protocols of animal models

This animal study was approved by the Animal Ethical Care Committee of Qiqihar Medical University (Approval No.: QMU-AECC-2024-174). Thirty 6-week-old SPF-grade male SD rats, weighing (220 ± 20) g, were purchased from Liaoning Changsheng Biotechnology Co., Ltd. (Animal License No.: SYXK(Hei) 2021-013). The rats were housed in the Animal Experiment Center of Qiqihar Medical University under a temperature of 25 °C, humidity of 45%, and a 12-h light-dark cycle, with free access to water and food.

After 1 week of acclimatization, 24 male Sprague-Dawley rats (n = 6) were randomly selected and administered 250 mg/kg adenine (Yuanye Bio-Technology Co., Ltd., Shanghai, China) suspension via oral gavage once daily for three consecutive weeks to establish the RIF model (Luo et al., 2022). Throughout the experimental period, animals were provided *ad libitum* access to standard rodent chow and water. The RIF rats were randomly divided into 4 groups: model group, Losartan group (LST) (Yuanye Bio-Technology Co., Ltd., Shanghai, China),low-dose AECS group (AECSL) and high-dose AECS group (AECSH), with 6 rats in each group. Body weight was measured every 2 weeks for each group. After successful modeling was confirmed by histopathological staining, the AECSL and AECSH groups were administered 150 and 300 mg/kg/day, respectively, the LST group was administered 50 mg/kg/day, and the sham and model groups were administered an equal amount of 0.9% NaCl, with gavage intervention for 4 weeks.

#### 2.8.2 Renal function analysis

Following the 4-week treatment, 24-h urine volume were collected using metabolic cages. The rats were restrained and anesthetized under sterile conditions. Blood was drawn from the abdominal aorta of the rats in each group. Renal function indicators serum creatinine (Scr), urine creatinine (Ucr), blood urea nitrogen (BUN) were assessed with an enzyme marker apparatus (SpectraMax iD3, CA, United States), according to the methods outlined in the creatinine and blood urea nitrogen assay kits (Jiancheng Bioengineering Institute, Nanjing, China).

#### 2.8.3 Body weight and renal index

Upon completion of blood collection, the abdominal cavity was incised layer by layer, with the costovertebral angle serving as a landmark. The tissues surrounding the kidney were meticulously separated, and the kidney was extracted through the incision. The renal pedicle was clamped and ligated to prevent bleeding. Subsequently, the kidney was removed, and the fat and fascia were swiftly excised. Surface body fluids were wiped with filter paper. The corresponding renal index was then calculated. The calculation formula was: Renal index (%) = Kidney weight(g)/Body weight(g)×100.

#### 2.8.4 Renal pathological and fibrosis level assessment

Kidney tissues were fixed in 10% paraformaldehyde solution and then washed with running water overnight. Standard procedures for dehydration, clearing, infiltration, embedding, sectioning, and mounting were performed using a paraffin slicer (Leica HistoCore MULTICUT, Germany) and tissue embedding machine (Leica HistoCore Arcadia H, Germany). The resulting sections were stained with hematoxylin and eosin (HE) and Masson’s trichrome (Masson) stain. HE-stained sections were examined under a light microscope to assess morphological changes in the renal tissue, while Masson-stained sections were analyzed to evaluate the extent of renal fibrosis. Fibrotic area analysis avoids vascular and glomerular areas. Semiquantitative analysis of renal damage was performed using Image Pro Plus 6.0 software.

#### 2.8.5 Evaluation of toxicity

Male Sprague-Dawley rats (n = 6) were randomly allocated into sham control (equivalent volume of 0.9% NaCl), low-dose AECS (150 mg/kg/day), and high-dose AECS (300 mg/kg/day) groups, with all treatments administered via oral gavage for 7 consecutive days. Hepatic, pulmonary, and cardiac tissues were collected and subjected to H&E staining to assess dose-dependent effects of CS extract administration.

#### 2.8.6 Immunohistochemistry

Immunohistochemical techniques were utilized to assess the expression of α-SMA and fibronectin proteins (diluted 1:100, Proteintech, China) in rat kidney cortex sections. Initially, the sections were deparaffinized in xylene and subjected to gradient ethanol dehydration. Antigen retrieval was subsequently performed using citrate buffer, followed by washing with phosphate-buffered saline (PBS). Endogenous peroxidase activity was then inhibited using a 3% hydrogen peroxide solution, and the sections were blocked with bovine serum albumin. Primary antibodies against α-SMA and fibronectin were applied and incubated overnight at 4 °C, followed by the addition of corresponding secondary antibodies (diluted 1:100) with incubation at room temperature for 1 h. Following DAB staining, hematoxylin counterstaining, dehydration in ethanol, and clearing in xylene, the sections were mounted with neutral resin. Finally, the samples were examined under a microscope, and the brown-positive protein expression was quantitatively analyzed.

#### 2.8.7 Double immunofluorescence

Paraffin-embedded kidney sections were dewaxed, rehydrated through an ethanol gradient, and antigen-retrieved, then washed with PBS and blocked at room temperature for 15 min. Sections were incubated overnight at 4 °C with diluted primary antibodies CD68 or CD206 (1:200, Boster, United States), washed with PBS, and incubated with fluorescent-labeled secondary antibodies for 1 h at room temperature. After PBS washing, blocking was performed with normal goat serum, and the α-SMA primary antibody was applied overnight at 4 °C. Following PBST washing, HRP-conjugated secondary antibody was added for 30 min at room temperature. The sections were washed with PBS, counterstained for nuclei, rinsed with PBST, and mounted with anti-fade mountant. Images were acquired using an upright microscope (Nikon, Germany).

#### 2.8.8 RT-qPCR analysis of renal mRNA expression in rats

Total RNA was extracted from kidney tissues, reverse transcribed into cDNA, and amplified by RT-qPCR using SYBR Green Supermix. Glyceraldehyde-3-Phosphate Dehydrogenase (GAPDH) served as the internal reference, with three biological replicates. Relative quantification was performed using the 2^−ΔΔCt^ method. Primer sequences were listed in Table 1.

**TABLE 1:** Primer sequences for RT-qPCR.

Gene	Primer sequence (5′–3′)	
	Forward	Reverse
Akt1	GGC​AGG​AGG​AGG​AGA​CGA​TGG	TTC​ATG​GTC​ACA​CGG​TGC​TTG​G
Egfr	CAC​TAC​GCC​GCC​TGC​TTC​AAG	ACT​GTG​CCA​AAT​GCT​CCT​GAA​CC
Il-10	CCC​TGG​GAG​AGA​AGC​TGA​AGA​CC	CAC​CTG​CTC​CAC​TGC​CTT​GC
1l-6	ACT​TCC​AGC​CAG​TTG​CCT​TCT​TG	TGG​TCT​GTT​GTG​GGT​GGT​ATC​CTC
Gapdh	ACG​GCA​AGT​TCA​ACG​GCA​CAG	CGA​CAT​ACT​CAG​CAC​CAG​CAT​CAC

#### 2.8.9 Statistical analysis

Each experiment was independently repeated at least three times. Data were expressed as mean ± SEM and analyzed statistically using GraphPad Prism 10.1.2 software, which was also used for image processing. Normally distributed data were assessed by one-way ANOVA, while non-normally distributed data were analyzed using the nonparametric Wilcoxon rank-sum test. A P value of less than 0.05 was considered statistically significant.

## 3 Results

### 3.1 Chemical profiling of AECS by UHPLC-Q-Exactive Orbitrap-MS

**FIGURE 1:**
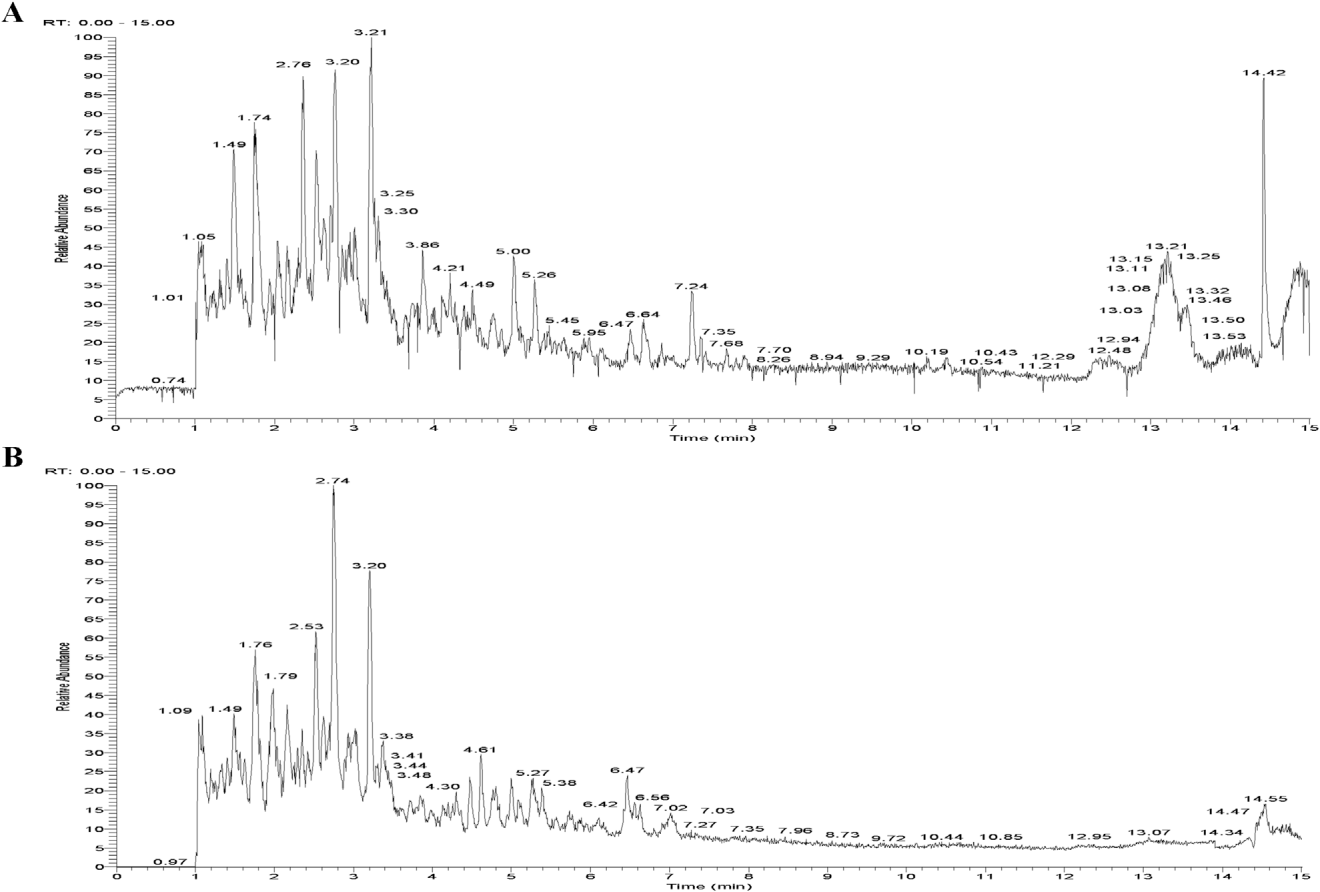
Total Ion Chromatogram of the AECS in different ionization modes. **(A)** Positive ion mode. **(B)** Negative ion mode.

A total of 64 compounds were identified through dual ionization mode analysis, comprising 35 flavonoids; 8 glycosides; 3 organic acids; 4 terpenoids; 4 coumarins; 3 alkaloids; 2 amino acids and derivatives; with 5 additional compounds classified as miscellaneous. Corresponding ion chromatograms for both positive and negative modes are presented in Figures 1A,B, while compound-specific data including retention times and mass spectra are detailed in Supplementary Table S1.

**FIGURE 2:**
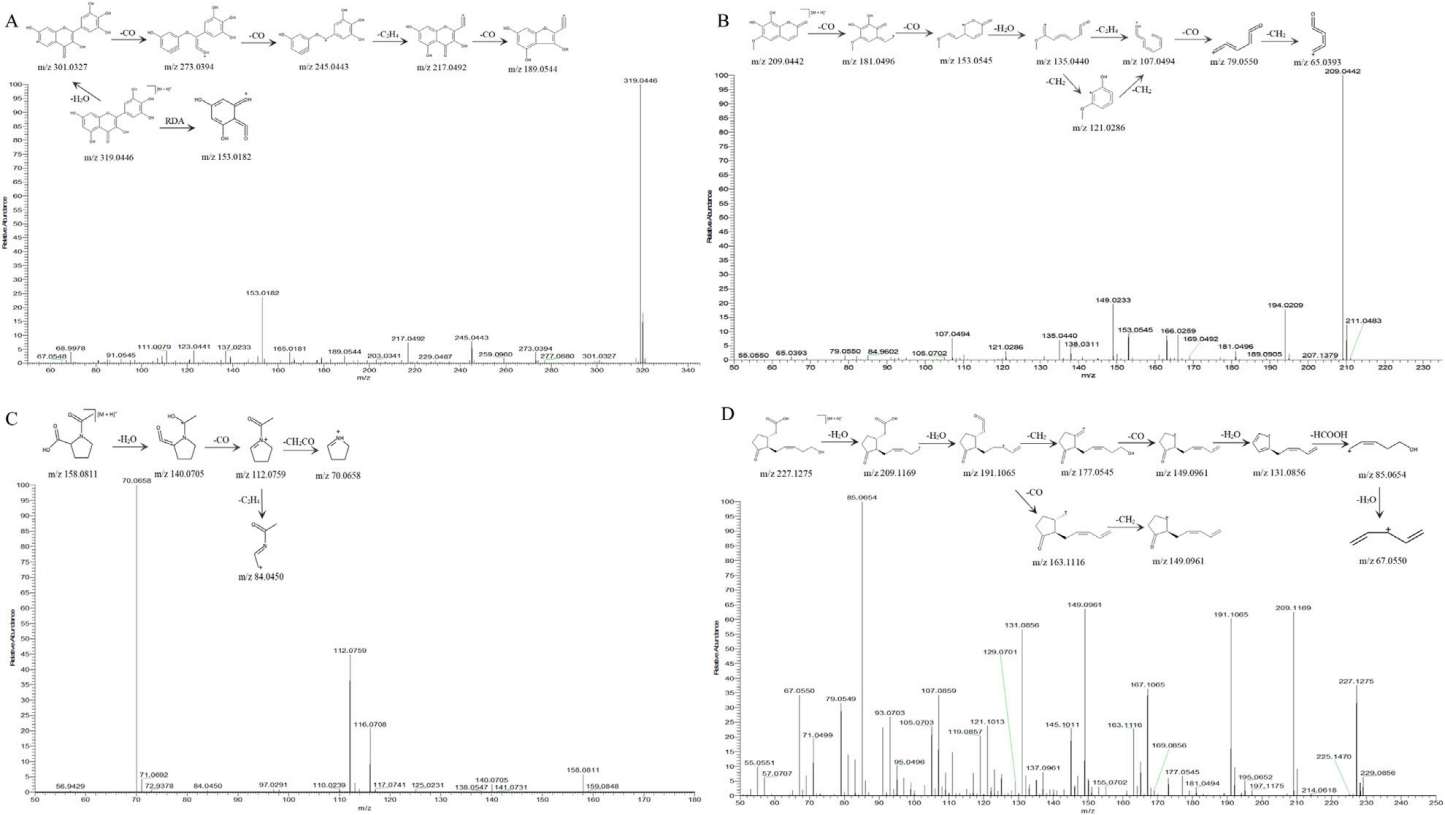
MS^2^ spectra and possible fragmentation pathway of **(A)** Myricetin. **(B)** Fraxetin. **(C)** 1-acetylprolin. **(D)** 12-hydroxyjasmonic acid.

Structural elucidation of fragmentation pathways was performed for representative com pounds spanning multiple classes: Myricetin (flavonoid), Fraxetin (coumarin), 1-Acetylprolin (Amino acid), and 12-hydroxyjasmonic acid (Organic acids), with detailed mechanistic analysis illustrated in Figure 2. Compound 25 exhibited a quasi-molecular ion at m/z 319.0446 [M + H]^+^ in positive ion mode, corresponding to the molecular formula C_15_H_10_O_8_. Two primary fragmentation pathways were observed:Retro-Diels-Alder (RDA) cleavage of the C-ring led to loss of C_8_H_6_O_4_, giving m/z 153.0182 [M + H-C_8_H_6_O_4_]^+^, while sequential loss of H_2_O produced m/z 301.0327 [M + H-H_2_O]^+^, followed by consecutive CO losses yielding m/z 273.0394 [M + H-H_2_O-CO]^+^ and m/z 245.0443 [M + H-H_2_O-2CO]^+^.Further fragmentation of m/z 245.0443 via C_2_H_4_ elimination followed by CO loss produced m/z 217.0492 and m/z 189.0544, respectively. The compound was conclusively identified as Myricetin by comparison with spectral databases and supported by literature (Wang et al., 2024).

Compound 16 exhibited a quasi-molecular ion peak at m/z 209.0442 [M + H]^+^ in positive ion mode, corresponding to the molecular formula C_10_H_8_O_5_. The precursor ion sequentially lost two CO molecules to generate m/z 181.0496 [M + H-CO]^+^ and m/z 153.0545 [M + H-2CO]^+^, followed by elimination of H_2_O yielding m/z 135.0440 [M + H-2CO-H_2_O]^+^. This intermediate further fragmented via either C_2_H_4_ loss or two consecutive CH_2_ losses to produce m/z 107.0494 [M + H-2CO-H_2_O-C_2_H_4_]^+^. Subsequent CO and CH_2_ eliminations resulted in terminal fragment ions at m/z 79.0550 [M + H-2CO-H_2_O-C_2_H_4_-CO]^+^ and m/z 65.0393 [M + H-2CO-H_2_O-C_2_H_4_-CO-CH_2_]^+^. Comparative analysis with mass spectral databases and literature (Lee et al., 2024) confirmed the identity of compound 16 as Fraxetin.

In positive ion mode, the quasi-molecular ion of compound 7 was observed at m/z 158.0811 [M + H]^+^, corresponding to the molecular formula C_7_H_11_NO_3_. The fragmentation pathway was characterized by sequential eliminations: initial H_2_O loss yielded m/z 140.0705 [M + H–H_2_O]^+^, followed by CO elimination from the dehydrated ion to generate m/z 112.0759 [M + H–H_2_O–CO]^+^. Subsequent fragmentation diverged into two distinct pathways:C_2_H_4_ loss produced m/z 84.0450 [M + H–H_2_O–CO–C_2_H_4_]^+^, CH_2_CO cleavage resulted in m/z 70.0658 [M + H–H_2_O–CO–CH_2_CO]^+^. The combined evidence from high-resolution mass spectral database matching and literature precedent (Song et al., 2022) conclusively identified compound 7 as 1-acetylprolin.

Compound 21 exhibited a quasi-molecular ion peak at m/z 227.1276 [M + H]^+^ in positive ion mode, corresponding to the molecular formula C_12_H_18_O_4_. Sequential losses of two H_2_O molecules generated fragment ions at m/z 209.1169 [M + H-H_2_O]^+^ and m/z 191.1065 [M + H-2H_2_O]^+^. The m/z 191.1065 ion fragmented through two distinct pathways: CO loss yielded m/z 163.1116 [M + H-2H_2_O-CO]^+^, Subsequent CH_2_ loss produced m/z 149.0961 [M + H-2H_2_O-CO-CH_2_]^+^; Initial CH_2_ loss generated m/z 177.0545 [M + H-2H_2_O-CH_2_]^+^, CO removal formed m/z 149.0961 [M + H-2H_2_O-CH_2_-CO]^+^, H_2_O elimination resulted in m/z 131.0856 [M + H-3H_2_O-CH_2_-CO]^+^, HCOOH loss yielded m/z 85.0654 [M + H-3H_2_O-CH_2_-CO-HCOOH]^+^, Final H_2_O removal produced m/z 67.0549 [M + H-4H_2_O-CH_2_-CO-HCOOH]^+^. The fragmentation pattern matched high-resolution spectral databases and literature reports (Brückel et al., 2024), confirming the identity of compound 21 as 12-hydroxyjasmonic acid.

### 3.2 Network pharmacology of CS for the treatment of RIF

#### 3.2.1 Prediction of potential targets for RIF treatment with CS

**FIGURE 3:**
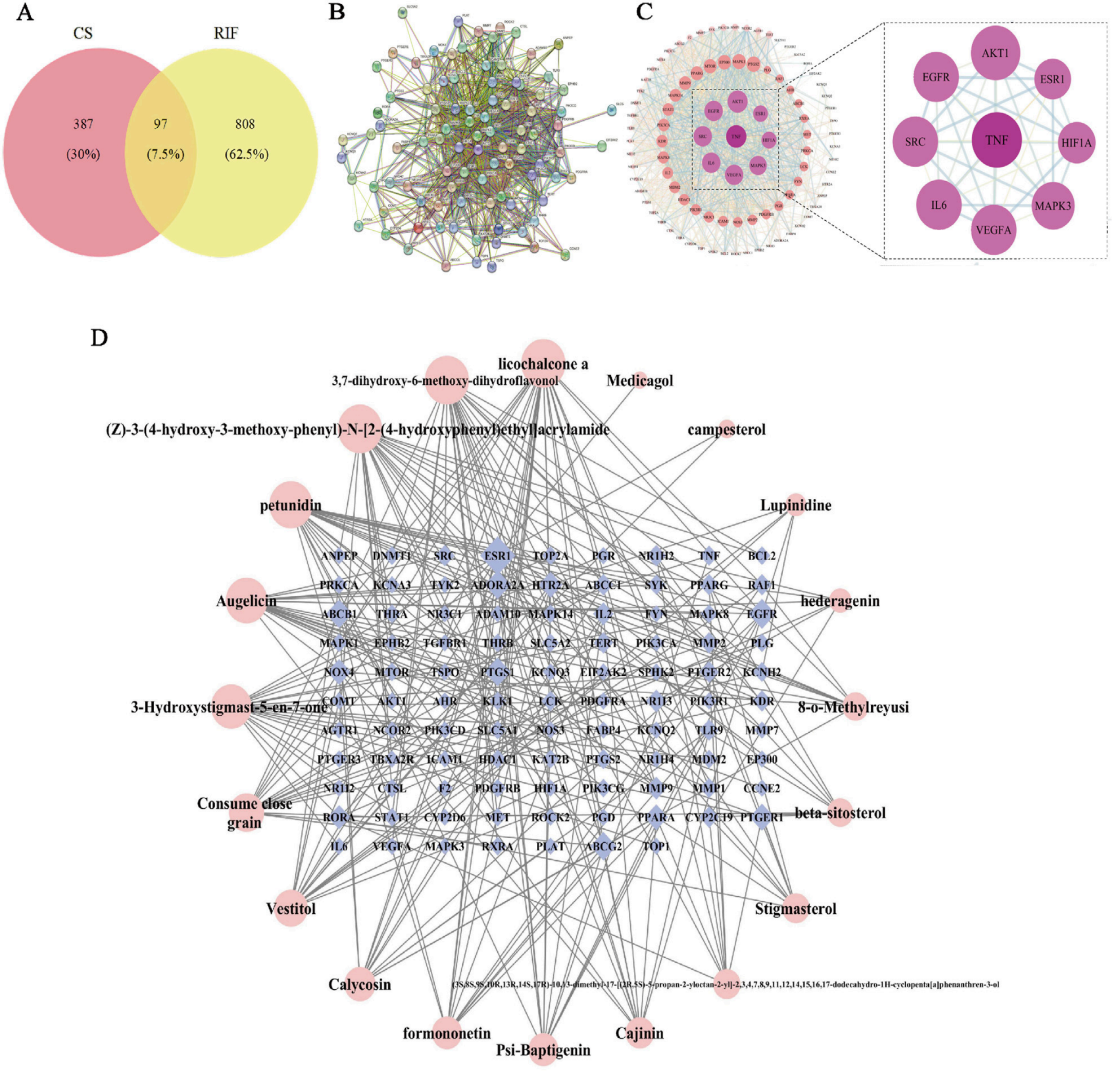
Network pharmacological analysis of CS in RIF. **(A)** Venn diagram of potential tar get of CS for RIF treatment. **(B)** Target protein PPI network analysis. **(C)** Visual analysis of PPI network. **(D)** Drugs- Active Ingredient-Target visualization network.

Based on predictions from the UHPLC-Q-Exactive Orbitrap-MS and TCMSP database and supplemented by literature, 20 valid active ingredients were identified after deduplication. Using the Swiss Target Prediction database, 484 potential target proteins for CS were predicted. These targets were subsequently entered into OMIM, STRING, GENE CARDS, DisGeNET, and DrugBank databases. After integration and removal of duplicates, a total of 905 RIF disease targets were obtained. A Venn diagram was generated using the EVenn platform, and 97 common targets were identified (Figure 3A).

#### 3.2.2 “Drugs-active Ingredient-Target“ visualization network

The data for drugs, active ingredients, and key targets were matched to construct the in teraction network. In the diagram, bluish violet diamonds represent the key targets of CS affecting RIF, while pink circles denote the active ingredients of the drugs. The results (Figure 3D) illustrate the complex interrelationships between“drug-active ingredient-target”, reflecting the intricate biological mechanisms of drug action in the body. The main active ingredients were identified as licochalcone A, 3,7-dihydroxy-6-methoxy-dihydroflavonol, petunidin, augelicin, and 3-Hydroxystigmast-5-en-7-one. These ingredients are suggested to be the key active ingredients for CS in the treatment of RIF.

#### 3.2.3 Screening of CS-RIF co-targets

The 97 key targets were imported into the STRING database to obtain a PPI network rela tionships (Figure 3B). These relationships were subsequently visualized in Cytoscape, resulting in the construction of a protein interaction network diagram (Figure 3C). The network topology parameters were analyzed based on the target protein interaction network. TNF, AKT1, EGFR, and SRC were identified as the top four core targets ranked by node degree in the PPI network, suggesting that they may be critical targets for CS in the treatment of RIF.

#### 3.2.4 GO and KEGG enrichment analysis

**FIGURE 4:**
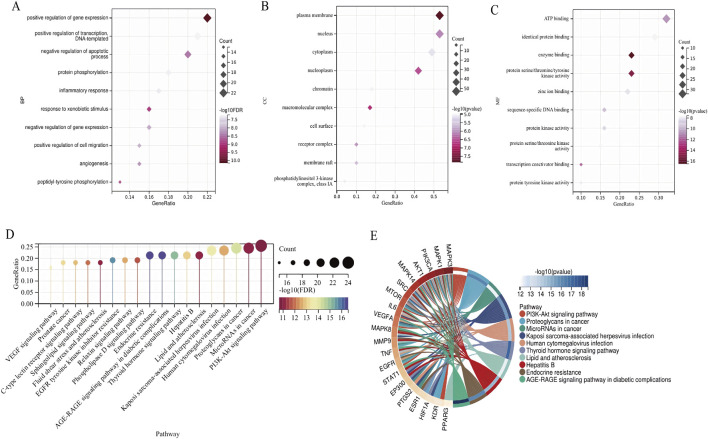
GO and KEGG enrichment analysis. **(A)** BP. **(B)** MF. **(C)** CC. **(D)** KEGG enrichment analysis of CS action targets. **(E)** The complicated relationship between target genes and KEGG pathways in CS.

The DAVID database was utilized to perform GO and KEGG enrichment analysis on the intersected drug-disease targets obtained. The top 10 terms for BP, CC, and MF, along with 18 KEGG pathways, were selected for visualization (*P* < 0.05). The GO analysis indicated (Figures 4A–C) that CS primarily participated in biological processes such as positive regulation of gene expression and protein phosphorylation; it involved cellular components like the plasma membrane and nucleus; and influenced molecular functions including ATP binding and identical protein binding. The KEGG analysis results showed (Figures 4D,E) that CS likely exerted its anti-RIF effects through signaling pathways such as PI3K-Akt, Proteoglycans in cancer, AGE-RAGE, and EGFR.

#### 3.2.5 Molecular docking verification

**FIGURE 5:**
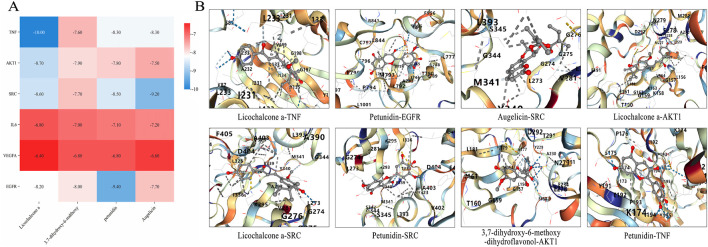
Molecular docking pattern diagram of core components of AECS. **(A)** Thermogram of binding energy of active ingredients and key targets (kcal/mol). **(B)** Molecular docking verification of active ingredient and key target.

To further investigate the mechanism by which CS treats RIF, molecular docking was conducted between the active ingredients of CS and the top-ranked targets in the PPI network based on Degree value. The binding energies between the core ingredients and their targets were predicted, and binding affinities were analyzed (Figure 5A). The results demonstrated (Figure 5B) that all binding energies were below −5.0 kcal/mol, indicating a strong binding affinity between CS and the targets, which was consistent with predictions from the network pharmacology platform. It was further predicted that CS primarily acts through these targets and active ingredients to exert its effects. The findings were deemed reliable.

### 3.3 Experimental verification that CS has anti-RIF effect

#### 3.3.1 CS protects renal function

**FIGURE 6:**
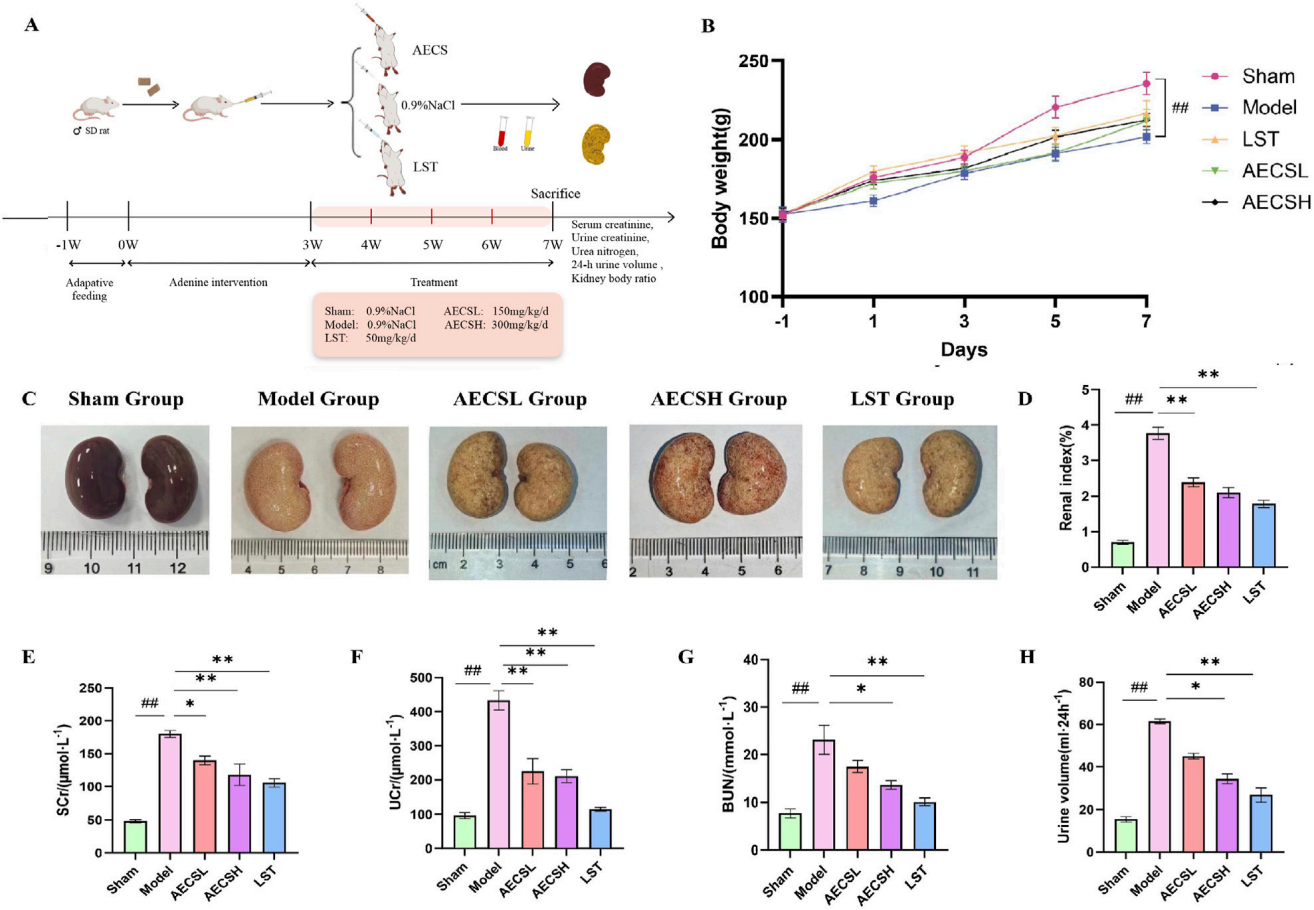
CS can effectively delay against the deterioration of renal function in RIF rats. **(A)** Experimental schedule (n = 6). **(B)** Rat body weights. **(C)** Macroscopic view of the kidney. **(D)** Changes in renal index. **(E)** Changes in SCr. **(F)** Changes in UCr. **(G)** Changes in BUN. **(H)** 24-h urine volume.^*##*^ *p* < 0.01 vs. the sham group; ^*^ *p* < 0.05 vs. the model group; ^**^ *p* < 0.01 vs. the model group.

Renal function is pivotal in assessing the efficiency of drug metabolism and excretion. The preparation of SD rat models for RIF disease and the administration plan for AECS were depicted in Figure 6A. Compared to the sham group, the model group exhibited significantly elevated levels of SCr, UCr, BUN, and 24-h urine volume (Figures 6E–H, *P* < 0.01). Conversely, the LST group and each subgroup of AECS demonstrated significantly reduced levels of SCr, UCr, BUN and 24-h urine volume (*P* < 0.01) compared to the modelgroup. Among the AECS dose groups, the high-dose group exhibited the most effective results, indicating that AECS effectively ameliorated the occurrence and progression of RIF.

#### 3.3.2 Effects of CS on body weight and renal index

Compared to the sham group, the body weight of the model group was significantly reduced (Figures 6B *p* < 0.01), accompanied by late-stage symptoms such as mental lethargy, decreased food intake, pale auricles, and dry body hair. The renal index (Figure 6D) was significantly elevated in the model group compared to the sham group (*P* < 0.01). In contrast, the LST group and each subgroup of AECS exhibited a significantly reduced renal index (*P* < 0.01) compared to the model group. These findings suggest that AECS may ameliorate the slow weight gain induced by adenine in rats and partially delay the deterioration of renal tissue.

#### 3.3.3 CS alleviates renal pathological and fibrosis

The evolution of renal tissue pathology is a crucial component in verifying the pharmacological mechanisms underlying drug therapeutic effects. As shown in Figure 6C, the kidneys in the model group were visibly enlarged and appeared pale with an irregular surface and nodular or granular changes, in contrast to the deep red kidneys of the sham group. In comparison, the aforementioned symptoms were improved to varying degrees in the treatment groups relative to the model group.

**FIGURE 7:**
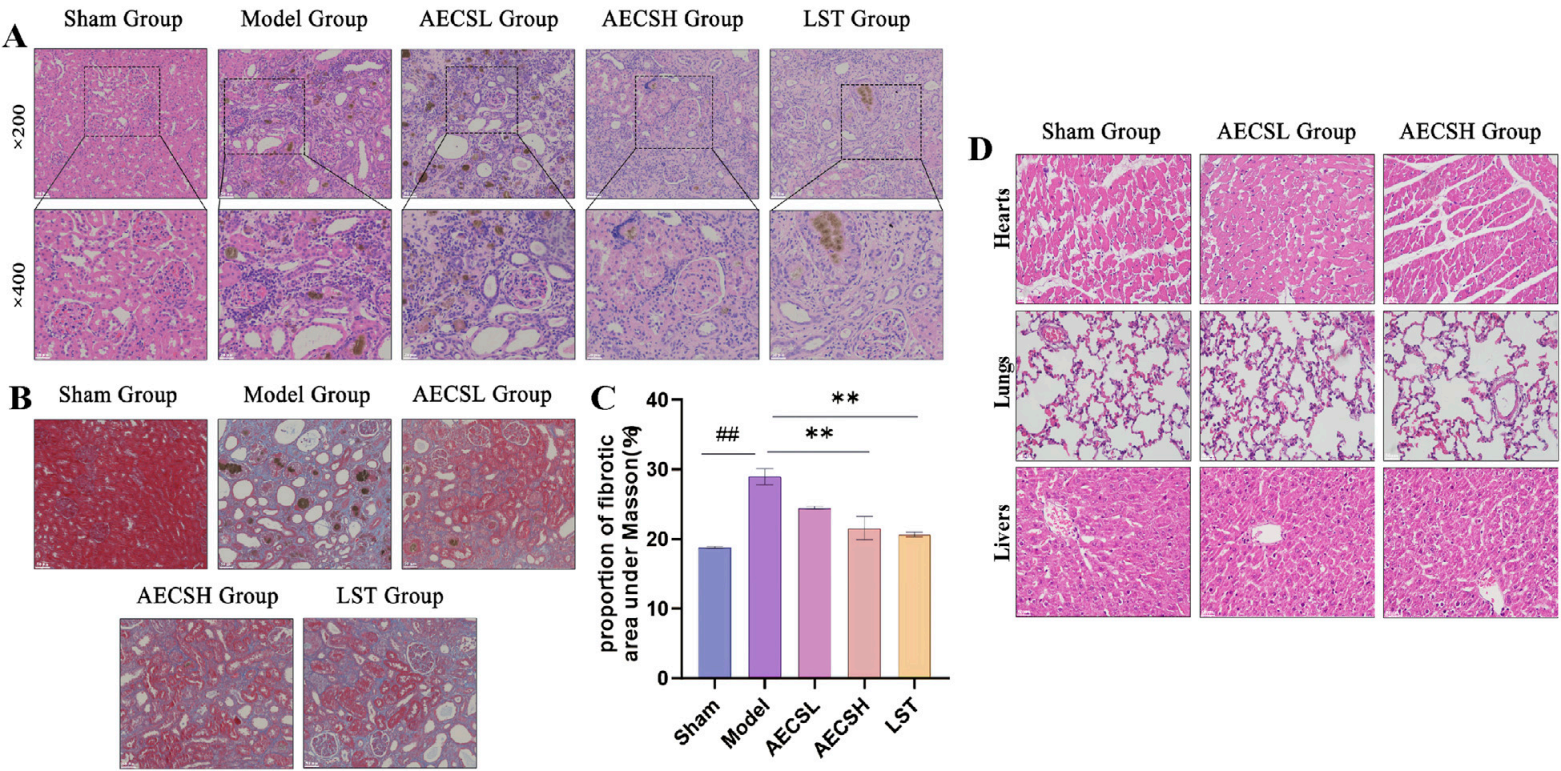
CS alleviates renal pathological and fibrosis in RIF rats. **(A)** Representative photomicrographs of H&E-stained renal pathological tissue, scale bars (20, 50 μm). **(B)** Representative photomicrographs of Masson-stained renal pathological tissue, scale bar (50 μm). **(C)** Quantitative Analysis of Fibrotic Area Proportion under Masson (n = 4). **(D)** Toxicity assessment of AECS in RIF rat models was performed through histo-pathological examination of hepatic, pulmonary, and cardiac tissues using H&E stain-ing (50 μm). ^*##*^ *p* < 0.01 vs. the sham group; ^**^ *p* < 0.01 vs. the model group.

HE staining results (Figure 7A) revealed that renal units in the sham group maintained a normal structure without significant pathological damage, whereas the model group exhibited urate and hemosiderin deposition. The number of glomeruli was reduced, with glomerular atrophy and sclerosis observed. Renal tubules were dilated, and epithelial cells showed degeneration and necrosis. Extensive mononuclear and lymphocytic inflammatory infiltration was present in the renal interstitium, accompanied by fibrotic changes. In contrast, improvements in glomerular, tubular, and interstitial inflammatory infiltration and lesions were observed in the LST group and the AECS groups compared to the model group.

Masson staining results (Figure 7B) demonstrated minimal blue staining and collagen fiber deposition in the kidneys of the sham group. Conversely, extensive collagen fiber deposition and widespread fibrosis were found in the model group. Compared to the model group, varying degrees of reduction in collagen fiber deposition and fibrosis were observed in the LST group and the AECS groups. The positive fibrotic area in the kidneys of the model group was significantly increased compared to the sham group (*P* < 0.01). Conversely, the positive fibrotic area was significantly decreased in the LST group and the AECS groups compared to the model group (*P* < 0.01), as shown in Figure 7C. These findings further indicate that CS has an ameliorative effect on RIF.

#### 3.3.4 Toxicological assessment of AECS in RIF rats

H&E staining was performed on hepatic, pulmonary, and cardiac tissues, and no significant inflammation or histopathological changes were detected across all groups through histological examination (Figure 7D).

#### 3.3.5 CS treatment suppresses α-SMA and fibronectin protein expressions in RIF rats

**FIGURE 8:**
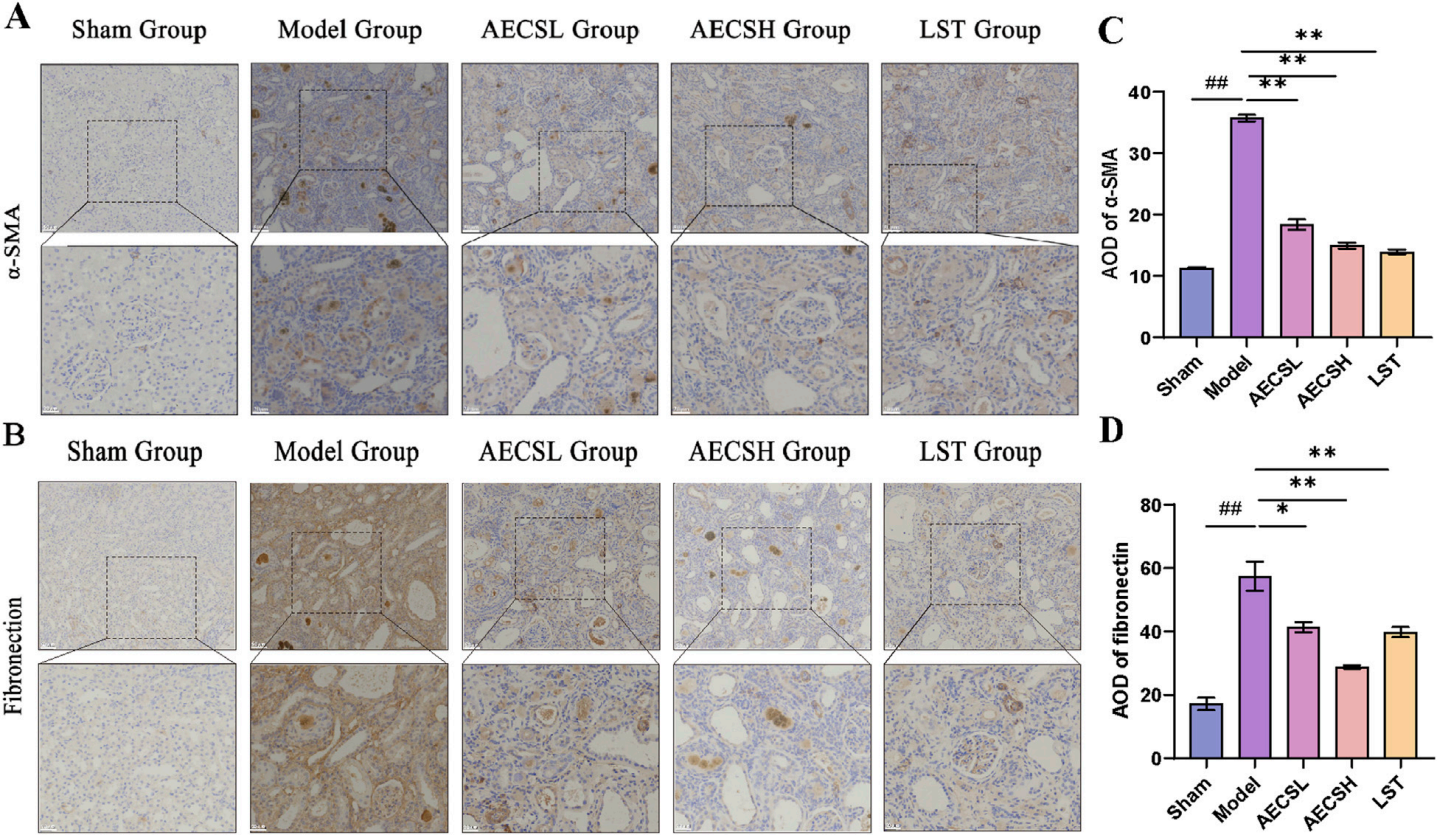
CS treatment suppresses α-SMA and Fibronectin protein expressions in RIF rats. **(A)** The expression of α-SMA was detected by Immunohistochemistry. **(B)** The expression of Fibronectin was detected by Immunohistochemistry. **(C)** Mean optical density levels of α-SMA in the kidneys. **(D)** Mean optical density levels of Fibronectin in the kidneys. (n = 4), ^*##*^ *p* < 0.01 vs. the sham group; ^*^ *p* < 0.05 vs. the model group; ^**^ *p* < 0.01 vs. the model group. Scale bars (20, 50 μm).

The pathological hallmarks of RIF include the upregulation of the interstitial marker α-SMA and the excessive deposition of the epithelial-mesenchymal transition (EMT)-related marker FN (Djudjaj and Boor, 2019). In the sham group, α-SMA and FN proteins were minimally expressed in the glomerular mesangial areas and renal interstitium of rats. Compared to the sham group, In comparison with the control group, the expression of α-SMA and FN in the renal tissues of the model group showed a significant increase (*P* < 0.01). However, both LST and AECS treatments markedly downregulated the expression of α-SMA and FN compared to the model group (*P* < 0.01). As shown in Figures 8A–D. These findings indicate that CS protects against RIF by concurrently suppressing EMT-driven fibrogenesis and pathological ECM deposition.

#### 3.3.6 CS attenuates MMT in RIF rats

**FIGURE 9:**
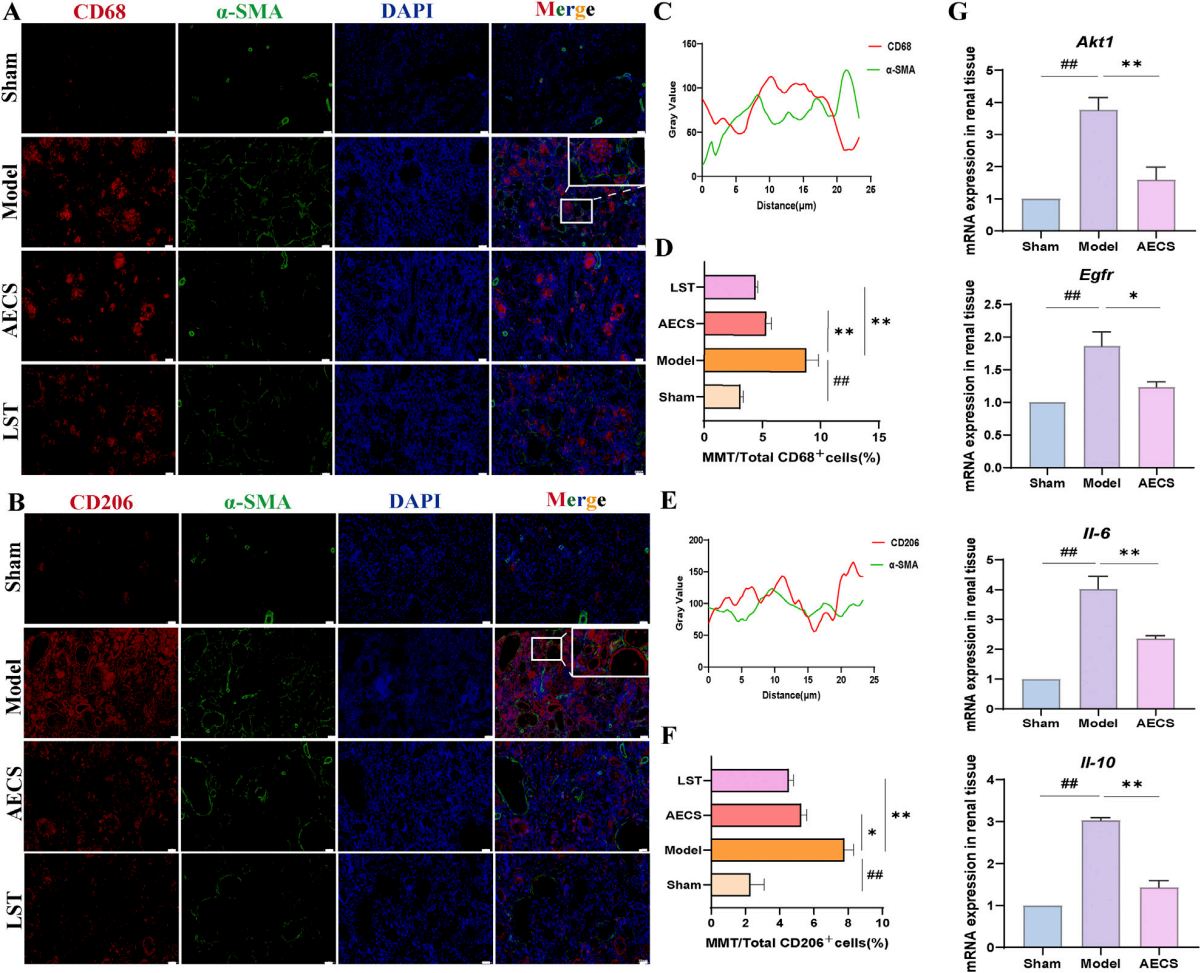
CS attenuates MMT in RIF rats. **(A)** Co-staining of the α-SMA (green) with CD68 (red) by Double Immunofluorescence. **(B)** Co-localization analysis of CD68+α-SMA^+^ MMT cells. **(C)** Staining intensity of CD68+α-SMA^+^ MMT cells was quantified. **(D)** Co-staining of the α-SMA (green) with CD206 (red) by Double Immunofluorescence. **(E)** Co-localization analysis of CD206+α-SMA^+^ MMT cells. **(F)** Staining intensity of CD206+α-SMA^+^ MMT cells was quantified (n = 4). **(G)** mRNA Expression of AKT1, EGFR, IL-6, and IL-10 in RIF rats kidney (n = 3). ^*##*^ *p* < 0.01 vs. the sham group; ^*^ *p* < 0.05 vs. the model group; ^**^ *p* < 0.01 vs. the model group. Scale bars (40 μm, 10 μm).

MMT was closely associated with renal pathologies, with M2 macrophages playing a pivotal role in RIF (Li G. et al., 2024). Rat kidney tissues were co-stained for general macrophage markers CD68+α-SMA^+^(Figures 9A,C) and M2-specific markers CD206+α-SMA^+^(Figures 9B,E) using double immunofluorescence. Compared to the sham group, the model group exhibited significantly enhanced fluorescence intensity of MMT cells, particularly within M2 macrophages (*P* < 0.01). LST and AECS treatment markedly reduced MMT cells fluorescence intensity in both total (*p* < 0.05) and M2 (*P* < 0.01) macrophage populations as shown in Figures 9D,F.

#### 3.3.7 Effect of CS on mRNA Expression of *Akt1, Egfr, Il-6* and *Il-10* in RIF rats kidney

The network pharmacology-predicted core targets AKT1 and EGFR, along with inflammatory cytokines IL-6 and IL-10, were experimentally validated. Compared to the sham group, the model group exhibited significantly elevated mRNA levels of *Akt1, Egfr, Il-6* and *Il-10* in renal tissues (*P* < 0.01). AECS treatment significantly decreased the expression of *Akt1, Egfr, Il-6* and *Il-10* compared to the model group (*P* < 0.01), as shown in Figure 9G.

## 4 Discussion

The pathogenesis of RIF is a multifactorial, multi-step, and multi-level complex process. Currently, no specific first-line clinical therapies are available for RIF. Existing treatments primarily include anti-inflammatory drugs (non-steroidal agents, corticosteroids, etc.), antihypertensive agents (diuretics, beta-blockers, etc.), and immunosuppressants (Li X. et al., 2024). Extensive evidence in recent years (Song L. et al., 2024; Song Z. Y. et al., 2024) has demonstrated that TCM hold significant potential in anti-fibrosis treatments. *Caulis spatholobi*, a TCM, has been shown to reduce fibrinogen levels, promote fibrinolysis, and improve diabetic nephropathy (Do et al., 2018). However, whether CS could serve as a potential effective treatment for RIF has not been reported, and its underlying therapeutic mechanism remains unclear.

In this study, we used a more efffcient and convenient UHPLC-Q-Exactive Orbitrap-MS method to identify 64 chemical components from AECS, mainly including flavonoids, glycosides, organic acids, terpenoids, alkaloids and coumarins. The findings elucidate the material basis of AECS’s medicinal effects, providing critical insights for further research on its active components. Concurrently, network pharmacology was employed to merge and deduplicate data, ultimately identifying 20 active ingredients. The primary active ingredients included licochalcone a, petunidin, augelicin, and calycosin. Licochalcone a was shown to ameliorate liver injury and its progression to fibrosis in Wistar rats by downregulating the PI3K/Akt/FoxO pathway (Zhou et al., 2021). Petunidin and augelicin demonstrated anti-inflammatory and antioxidant properties (Zheng et al., 2020). Calycosin significantly reduced RIF in diabetic rats by mitigating inflammation and oxidative stress (Elsherbiny et al., 2020). Furthermore, pretreatment of mesenchymal stem cells with calycosin was found to enhance the anti-RIF effect by inhibiting the TGF-β1/TNF-α/TNFR1/necroptosis pathway, thereby improving RIF in UUO mice and suppressing necroptosis (Hu et al., 2023).

A total of 97 key targets for the treatment of RIF using CS were identified. “Drug-active ingredients-target”and PPI visualization networks were constructed, with the top four core targets, ranked by node degree, being TNF, AKT1, EGFR, and SRC. TNF was found to promote fibroblast activation and RIF by inducing and releasing Indian hedgehog in renal epithelial cells (O'Sullivan et al., 2023). AKT1, an essential subfamily regulator within the AKT kinase family, played a critical role in preventing RIF occurrence and progression. In instances of insufficient or absent AKT1 expression, the expansion of RIF and dedifferentiation of renal tubular cells during the transition from acute kidney injury to CKD were significantly slowed (Kim et al., 2021). EGFR, with low expression in normal kidneys, was shown to be ubiquitinated and degraded by E3 ubiquitin ligase, thereby improving renal tubular epithelial cell injury and interstitial fibrosis (Zhu et al., 2020). SRC, a pivotal mediator in renal vascular endothelial-mesenchymal transition, inhibited AMBRA1-mediated mitophagy to exert its antifibrotic effect (Gui et al., 2024).

The key targets for CS treatment of RIF were analyzed using GO and KEGG analyses. GO functional enrichment results indicated that the therapeutic effect of CS on RIF likely involved the regulation of biological processes such as protein phosphorylation, ATP binding, and positive regulation of gene expression. KEGG pathway enrichment results demonstrated that core target genes were enriched in signaling pathways including PI3K/Akt, Proteoglycans in cancer, AGE/RAGE, and EGFR, suggesting that CS could exert its anti-RIF effect by targeting these biological processes and signaling pathways. To verify the reliability of these findings, molecular docking technology was employed to assess the binding abilities of effective active ingredients (licochalcone a, petunidin, etc.) and key targets (AKT1,EGFR, etc.), all of which exhibited binding energies less than −5.0 kcal/mol, confirming the reliability of the results.

**FIGURE 10:**
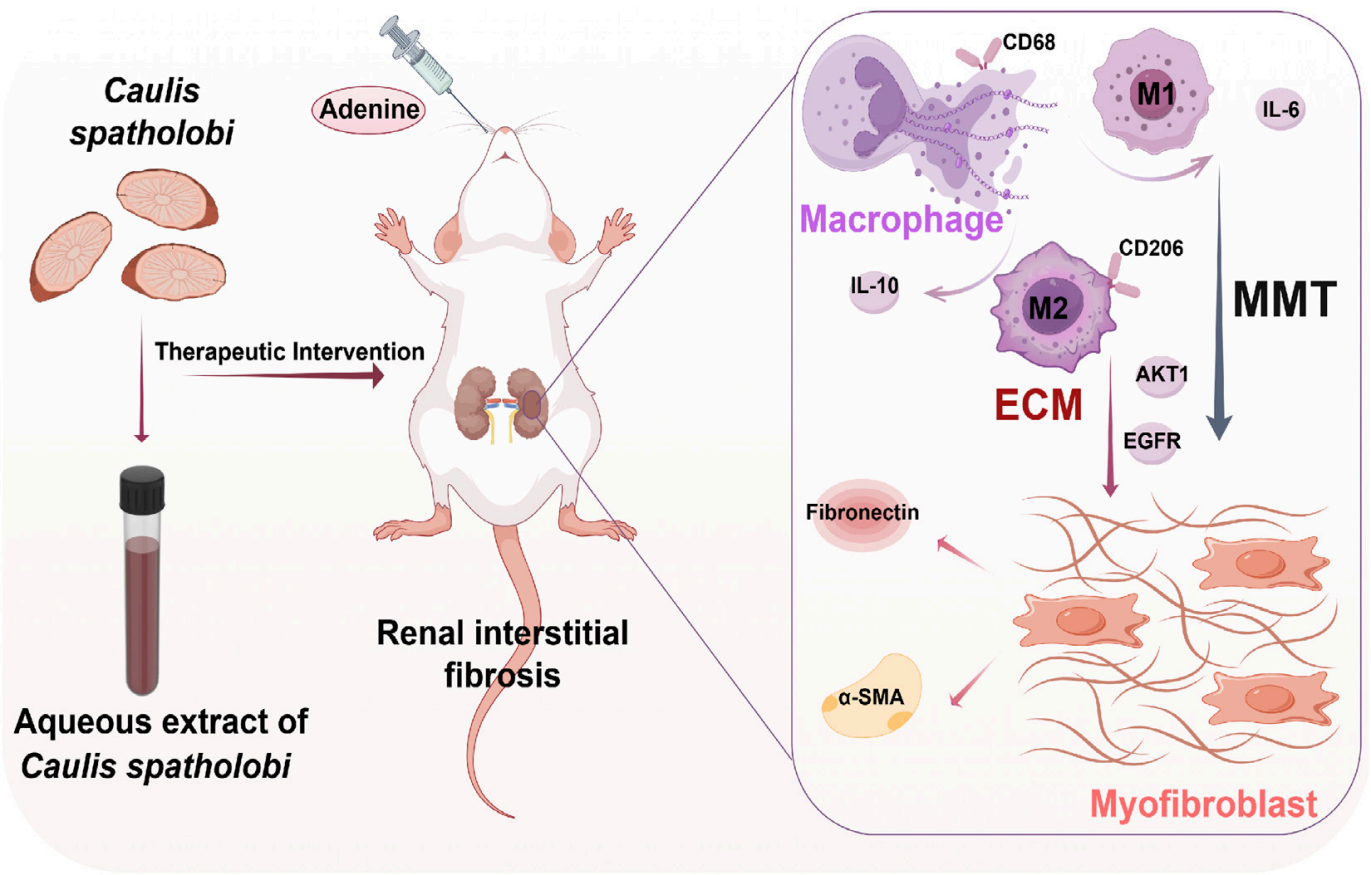
Aqueous extract of *Caulis spatholobi* demonstrates therapeutic potential against adenine-induced renal interstitial fibrosis in rats by alleviating histological disturbances, suppressing inflammatory responses, and inhibiting macrophage-myofibroblast transition. (created with Figdraw) MMT: macrophage-to-myofibroblast transition, ECM: extracellular matrix, CD68: cluster of differentiation 68,CD206: cluster of differentiation 206, α-SMA: alpha-smooth muscle actin,IL-6: interleukin-6, IL-10: interleu kin-10, AKT1: AKT serine/threonine kinase 1, EGFR: epidermal growth factor receptor.

An adenine-induced model of RIF in rats was established (Figure 10). Compared to surgical mod els such as unilateral ureteral obstruction (UUO), this model was simpler to operate and more closely resembled human CKD models, making it suitable for clinical functional endpoint evaluation (Jørgensen et al., 2024). Adenine, catalyzed by xanthine oxidase, generated 2,8-dihydroxyadenine, which deposited in the renal tubules, causing obstruction and damage to the nephrons, leading to RIF (Yamato et al., 2020). In this experiment, each drug-treated group could reduced kidney function indicators such as SCr, UCr, BUN, and 24-h urine volume levels, downregulated the expression of RIF marker proteins α-SMA and FN, thereby suppressing EMT-driven fibrogenesis and pathological ECM deposition. Moreover, compared to the AECSL group, the LST and AECSH groups (300 mg/kg/d) demonstrated more significant therapeutic effects.

MMT cells were demonstrated to contribute to multi-organ fibrosis, including renal (Ren et al., 2025), cardiac (Shen et al., 2025), hepatic (Xia et al., 2023), pulmonary (Peng et al., 2025), and epidural pathologies (Sun et al., 2025). Under chronic pathological conditions, macrophage infiltration and co-expression of CD68+α-SMA^+^and CD206+α-SMA^+^were observed to drive fibrotic progression via MMT cells differentiation (Liu T. et al., 2024). Early injury was dominated by M1 macrophages secreting pro-inflammatory cytokines such as IL-6 that exacerbated tissue damage, whereas late-stage repair involved M2 macrophages promoting abnormal ECM remodeling through anti-inflammatory such as IL-10 and pro-fibrotic cytokine release. This pathological fibrosis, characterized by diffuse interstitial hyperplasia, disrupted organ architecture, impaired function, and established a microenvironment perpetuating fibrotic progression (Yang et al., 2025). In our study, RIF rats in the model group exhibited upregulated expression of pan-macrophage marker CD68 and M2-specific marker CD206 compared to the sham group, with α-SMA co-expression indi-cating differentiation into MMT cells characterized by dual CD68+α-SMA^+^ or CD206+α- SMA^+^ immunophenotype. LST and AECS treatments significantly reduced MMT cells populations, with CD206+α-SMA^+^ MMT cells predominating over CD68+α-SMA^+^ MMT cells, underscoring the pivotal role of M2 macrophages in MMT-mediated RIF. Furthermore, These MMT-derived M1-type macrophages secreted pro-inflammatory cytokine IL-6, while M2-type macrophages produced anti-inflammatory IL-10, both of which were downregulated by AECS treatment. These findings provide experimental data and theoretical support for the treatment of RIF with CS.

Collectively, this study elucidated the pharmacodynamic basis and mechanism of AECS against RIF through integrated UHPLC-Q-Exactive Orbitrap-MS analysis, network pharmacology, molecular docking, and *in vivo* validation. It was demonstrated that AECS exerted protective effects primarily by suppressing MMT-mediated inflammatory responses, leading to downregulation of key targets including IL-6 and IL-10. Microscopic examination revealed no significant pathological alterations in hepatic, pulmonary, or cardiac tissues following AECS administration at 300 mg/kg/day, indicating an absence of overt organ toxicity within this dosage range. This favorable safety profile supports the potential applicability of AECS as an RIF intervention strategy. However, limitations were acknowledged: a) While cytokine IL-6 and IL-10 downregulation was mediated by MMT inhibition, the relationship between MMT and other network-predicted targets AKT1 and EGFR remains unverified; b) The research primarily remained at the animal level, with limited cell and clinical trials, necessitating deeper re-search to confirm the efficacy of CS. In our subsequent research, our team will conduct more in-depth studies based on these issues.

## 5 Conclusion

This study was based on UHPLC-Q-Exactive Orbitrap-MS, network pharmacology and molecular docking technology, predicting that *Caulis spatholobi* could act through multiple targets such as AKT1, EGFR and IL6, mediating biological processes such as protein phosphorylation and ATP binding, and regulating multiple signaling pathways such as PI3K/Akt, Proteoglycans in cancer, AGE/RAGE, and EGFR, thereby inhibiting the progression of RIF. *In vivo* animal experiments were conducted using an adenine-induced RIF rat model, with treatment administered using the pharmaceutical losartan and AECS at different doses. Preliminary verification showed that AECS could improve renal function, inhibit MMT, which in turn downregulated the key molecules IL-6 and IL-10, alleviate pathological damage, delay matrix deposition and remodeling, and inhibit the formation of RIF. Our findings demonstrate that AECS represents a promising natural medicine-derived candidate with significant translational potential. Future studies will focus on comprehensive toxicology and pharmacokinetic assessments to establish its safety profile prior to clinical evaluation. Concurrently, extraction processes will be optimized and synergistic therapeutic effects with standard anti-fibrotic agents will be evaluated. This work provides a critical foundation for the future development and clinical translation of AECS.

## Data Availability

The original contributions presented in the study are included in the article/Supplementary Materials. Further inquiries can be directed to the corresponding author.

## Abbreviations

MMT, macrophage-to-myofibroblast transition; TCM, traditional Chinese medicine; CS, Caulis spatholobi; AECS, aqueous extract of CS; ECM, RIF extracellular matrix renal interstitial fibrosis.
